# Advances in management of metabolic dysfunction-associated steatotic liver disease: from mechanisms to therapeutics

**DOI:** 10.1186/s12944-024-02092-2

**Published:** 2024-04-02

**Authors:** Yuxiao Jiang, Lili Wu, Xiaopeng Zhu, Hua Bian, Xin Gao, Mingfeng Xia

**Affiliations:** 1 Department of Endocrinology and Metabolism, Zhongshan Hospital and Fudan Institute for Metabolic Diseases, Fudan University, 180 Fenglin Rd, Shanghai, 200032 China; 2 Department of Integrated Medicine, Guangxi Medical University Cancer Hospital, Nanning, China; 3 Department of Endocrinology and Metabolism, Wusong Branch of Zhongshan Hospital, Fudan University, Shanghai, China

**Keywords:** Metabolic dysfunction-associated fatty liver disease, Hepatic lipid metabolism, Multisystem disease, Pathophysiology, Medications, Clinical trials

## Abstract

Metabolic dysfunction-associated steatotic liver disease (MASLD) is the leading cause of chronic liver disease that affects over 30% of the world’s population. For decades, the heterogeneity of non-alcoholic fatty liver disease (NAFLD) has impeded our understanding of the disease mechanism and the development of effective medications. However, a recent change in the nomenclature from NAFLD to MASLD emphasizes the critical role of systemic metabolic dysfunction in the pathophysiology of this disease and therefore promotes the progress in the pharmaceutical treatment of MASLD. In this review, we focus on the mechanism underlying the abnormality of hepatic lipid metabolism in patients with MASLD, and summarize the latest progress in the therapeutic medications of MASLD that target metabolic disorders.

## Introduction

According to an international expert proposal in 2020, non-alcoholic fatty liver disease (NAFLD) should be updated to metabolic dysfunction associated with fatty liver disease (MAFLD) [1]. Two of the seven criteria for metabolic dysfunction must be met for the patient to be diagnosed with MAFLD [2]. Recent multi-society Delphi consensus statements have replaced the nomenclature of NAFLD with metabolic dysfunction-associated steatotic fatty liver disease (MASLD) [3]. The diagnosis of MASLD includes evidence of hepatic steatosis along with at least one of the following five cardiometabolic criteria: the presence of overweight or obesity, impaired glucose regulation or type 2 diabetes, hypertension, increased plasma triglycerides, or decreased high-density lipoprotein cholesterol (HDL-c) [3]. The new nomenclature of MASLD not only emphasizes the critical role of systemic metabolic dysfunction in the pathogenic process leading to MASLD, but also enhances the clinicians’ awareness to the concomitant metabolic dysfunction in patients with MASLD.

The global prevalence of NAFLD is approximately 25% according to previous studies [4]. Since the use of the new nomenclature of MASLD, the nationwide prevalence of MASLD in the United States has been found to be 32.45% [5]. More recently, a community-based study among East Asians in Hong Kong showed that the prevalence of MASLD was 26.7%, and the difference between the prevalence of NAFLD and MASLD in the same population was minimal [6]. Other recent statistical data show that MASLD affects more than 30% of adults globally and causes a heavy economic burden of over $100 billion in the USA [7]. Therefore, the prevalence of MASLD is estimated to be 25–30%, similar to that of NAFLD. The estimated pooled all-cause mortality rate for patients with NAFLD was 12.60 per 1000 person years (PYs). This rate included 4.20 per 1000 PYs for mortality specific to cardiac disease, 2.83 per 1000 PYs for mortality specific to extrahepatic cancer, and 0.92 per 1000 PYs for mortality specific to liver disease [8]. In the latest third National Health and Nutrition Examination Surveys 1988–1994 (NHANES III) study including 13,856 individuals, patients with MASLD was proved to be associated with significantly higher all-cause mortality (adjusted HR 1.127, 95% CI 1.056–1.201) and diabetes-related mortality (adjusted HR 1.911, 95% CI 1.418–2.574) than those without during follow-up [6]. Therefore, patients with MAFLD/MASLD even have worse clinical outcomes than those with NAFLD but not metabolic dysfunction [9]. However, the concomitant metabolic disorders are often overlooked in patients with NAFLD, thus leading to many adverse cardiovascular and liver-related outcomes. In comparison, the diagnosis of MASLD requires the presence of metabolic dysfunction, and the excessive liver fat accumulation in MASLD specifically originates from the state of systemic metabolic dysfunction. It enables better risk stratification and personalized treatment of fatty liver disease [10]. In this review, we discussed the critical role of metabolic dysfunction in the development and progression of MASLD, and summarized the latest progress in the drug treatment of MASLD from the perspective of systemic metabolic dysfunction.

## Role of metabolic dysfunction in the pathophysiology of MASLD

Metabolic dysfunction refers to the presence of obesity, hyperglycemia, hypertension or dyslipidemia clinically. The primary histological characteristic of MASLD is hepatocellular steatosis, which is thought to be the hepatic manifestation of metabolic syndrome [11]. According to the classic "two-hit theory" of fatty liver, the first hit involves excessive hepatic lipid deposition, and the second hit activates inflammatory cascades and fibrogenesis in hepatocytes after that [12], which results in non-alcoholic steatohepatitis (NASH) assessed by NAS scores and liver fibrosis classified as F1 to F4 by Metavir scores. However, subsequent studies over the last two decades have demonstrated that the pathogenesis of MASLD is much more complex than the two hits, and the "multiple-hit theory” has been widely accepted that multiple risk factors, including insulin resistance, nutritional factors, and lipid metabolism disorders, act together with genetic (e.g., patatin-like phospholipase domain containing 3 (PNPLA3), transmembrane 6 superfamily 2 (TM6SF2), and membrane bound O-acyltransferase domain containing 7 (MBOAT7) gene variants) and epigenetic (e.g., DNA methylation, histone modification, and m6A RNA methylation) factors to induce liver steatosis and progress to NASH and liver fibrosis [13]. Multiple risk factors jointly contribute to the progression of MASLD with dynamic changes from hepatic steatosis and inflammation, nonlinear progression of fibrosis to the recompensation of NAFLD-related cirrhosis, and novel pathophysiological mechanisms, such as impaired partial collagen degradation and hepatocyte regeneration, vascular remodeling and systemic inflammation enhancement, which are involved in the updated natural course of MASLD [14]. However, the latest theories on the pathogenesis of MASLD have not changed the important role of hepatic lipid accumulation as the initial and critical stage of this disease.

**Fig. 1:**
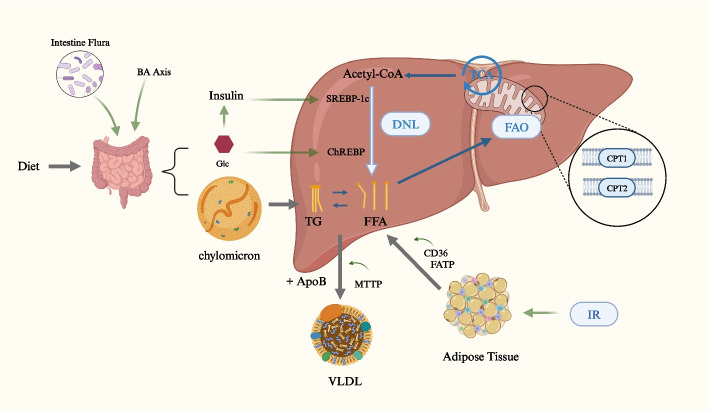
Overview of hepatic triglycerides metabolism. TG, triglycerides; SREBP, sterol regulatory element–binding protein; ChREBP, carbohydrate response element binding protein; FFA, free fatty acid; IR, insulin resistance; CD36, cluster of differentiation 36; FATP, fatty acid transport proteins; VLDL, very-low density lipoprotein; MTTP, Microsomal TG transfer protein; FAO, fatty acid β-oxidation; CPT, carnitine palmitoyl transferase; DNL, de novo lipogenesis

Hepatic fat accumulation arises when liver triglycerides acquisition exceeds removal. The mechanism of hepatic fat accumulation caused by metabolic dysfunction is shown in Fig. 1. Hepatic fat is derived from hepatic de novo lipogenesis (DNL), fatty acids released from the adipose tissue and dietary fat taken up in the intestine, and is metabolized through mitochondrial fatty acid β-oxidation (FAO) or exported out of the liver via very low density lipoprotein (VLDL) [15]. Metabolic dysfunction in any of the above hepatic lipid metabolism pathways could lead to MASLD.

### Insulin resistance (IR)

IR plays a pivotal role in the pathophysiology of metabolic syndrome, which might be crucial for the development of MAFLD [16] or MASLD [17]. Carbohydrate intake increases circulating insulin and glucose levels. In individuals with insulin resistance, postprandial glucose and insulin are usually higher than those in metabolically healthy individuals. In the liver, glucose and insulin act as important regulators of DNL and are discussed in the following section. Meanwhile, the impaired ability of insulin to suppress lipolysis in peripheral adipose tissue leads to excess release of free fatty acids (FFAs) and hyperlipidaemia, which promotes the uptake of FFAs and the accumulation of intrahepatic lipids [18, 19].

### Increased DNL

Insulin promotes the expression of sterol regulatory element–binding protein–1c (SREBP1c) [20], and glucose and fructose promote the translocation of carbohydrate response element binding protein (ChREBP) to the nucleus [21]. Both SREBP1c and ChREBP increase the expression of multiple enzymes that catalyze lipogenesis, including acetyl-CoA carboxylase (ACC), ATP citrate lyase (ACLY), fatty acid synthase (FAS), and stearoyl-CoA desaturase-1 (SCD1), as demonstrated in gene knockout mice [20, 21]. The states of cellular energy excess inhibit AMP-activated protein kinase (AMPK), a Ser/Thr protein kinase and an essential cellular energy sensor [22]. It has been recognized that AMPK activation inhibits DNL by down-regulating the level of ACC phosphorylation and SREBP1c expression [23]. Therefore, AMPK might be an important mediator that regulates hepatic lipogenesis under the metabolic dysfunction status. On the other hand, the excessive liver fat will in turn exacerbate IR through the production of excess ceramides and diacylglycerols (DAGs) [24]. Hepatic insulin resistance is caused by the activation of protein kinase Cε (PKCε) in high-fat diet mice due to an increase in hepatic plasma membrane sn-1,2-DAG content [25]. This inhibition of insulin receptor kinase (IRK) is the result of the interaction between hepatic DNL and IR, which creates a vicious cycle that aids in the development and progression of MASLD.

### Increased FFAs uptake

Chronic overnutrition is the fundamental reason of peripheral IR [24]. Circulating insulin functions to increase the uptake of fatty acids and enhance the synthesis of triglycerides in peripheral adipose tissue. On the other hand, in overfed individuals, high triglyceride accumulation triggers increased release of inflammatory factors such as tumor necrosis factor-α (TNF-α) and interleukin 6 (IL-6) as well as macrophage M1 activation in the adipose tissue [26]. Adipocytes in the periphery release FFAs more readily when low level of chronic inflammation and persistent stress in the adipose tissue activate stress-related signal transduction pathways such as inhibitor of kappa-B kinase beta (IKKB) and c-Jun N-terminal kinase (JNK) [27]. This results in the aberrant phosphorylation of insulin receptor substrate (IRS) and peripheral IR. Transporters like cluster of differentiation 36 (CD36) and fatty acid transport proteins (FATP) allow FFAs to enter hepatocytes [28]. In patients with metabolic dysfunction, the localization of CD36 as well as its palmitoylation level are significantly increased to facilitate the transport of FFAs into hepatocytes [29]. While inhibition of CD36 palmitoylation reduces its hydrophobicity, thus decreasing its localization on the plasma membrane and lipid rafts, and inhibiting hepatic FFAs uptake [30].

### Increased dietary fat and gut-liver axis

Dietary fat is absorbed in the intestine, packaged into chylomicrons and delivered into the systemic circulation. About 20% of the triglycerides in chylomicrons are delivered to the liver [31]. It is estimated that the common daily diet will furnish the liver with about 10 g of fat each day, while in individuals with high fat diet (such as typical American diet), the amount of fat entering the liver from daily diet doubles. Moreover, dietary fat, especially cholesterol, can modulate gut microbiota and bile acid profiles, thus driving the progression of MASLD [32]. The human gut is colonized by a large number of microorganisms. Alterations in the type and amount of gut microbiota are known as dysbiosis. Dysbiosis leads to the development and progression of MASLD through gut-liver axis that is regulated by bile acids (BA) receptors [33], such as farnesoid X receptor (FXR) and Takeda G-coupled protein receptor 5 (TGR5). Deactivation of FXR promotes DNL, and inhibits fatty acid oxidation (FAO) and VLDL triglycerides clearance [33], while TGR5 in small intestinal cells leads to the release of glucagon-like peptide 1 (GLP-1), which regulates food intake and glucose metabolism [34].

### Reduced mitochondrial FAO

Hepatic FAO and mitochondrial turnover are compromised in patients with MASLD [35]. It is necessary for carnitine palmitoyl transferase (CPT) to allow fatty acids to enter mitochondria. CPT1 and CPT2 are found in the two layers of the mitochondrial membrane respectively. CPT is reportedly upregulated [36] and CPT2 is inhibited [37] in patients with MASLD. Overexpression of CPT1A enhances hepatic FAO and lipid autophagy, thus reducing hepatic steatosis in high-fat-diet mice [38]. The expression of CPT1 is regulated by peroxisome proliferator-activated receptor (PPAR)-α [39], while the CPT2 expression is decreased in FXR deficiency, thus leading to the increase of SREBP1c-mediated FAS expression [40]. A typical example on the close correlation between mitochondrial dysfunction and MASLD is the MASLD patients carrying homozygous PNPLA3 I148M variant. The most potent genetic risk factor for MASLD is the PNPLA3 I148M variant [41]. Protein accumulation on lipid droplets inhibits the activity of adipose triglyceride lipase (ATGL), which leads to the accumulation of triglycerides in hepatic lipid droplets and a subsequent decrease in hepatic FAO [42]. Under fasting or ketogenic conditions, there is a decrease in I148M protein levels, which can cause excess hepatic triglyceride lipolysis and increase mitochondrial redox state; this can inhibit hepatic citrate synthase flux and ultimately result in liver injury [43].

### Abnormal VLDL secretion

Hepatocytes export excess triglycerides into the circulation by secreting apolipoprotein B-100 (ApoB100) containing very low density lipoprotein (VLDL) [44]. It has been suggested that the VLDL secretion is increased in patients with MASLD, but does not counteract the accumulation of excess triglycerides (TGs) [45]. Impairment in the VLDL secretion can lead to the development of MASLD in individuals with relatively good metabolic status. In TM6SF2 knockout mice, TG and cholesterol contents in VLDLs secreted into the blood were significantly reduced, which contributed to the accumulation of lipids in the liver [46, 47]. Similarly, in individuals with the TM6SF2 E167K variant, the VLDL assembly is inhibited, thus leading to the accumulation of hepatic fat and reduction in plasma TG concentrations [48]. As a result, MASLD patients with the TM6SF2 E167K mutation have a more reduced risk of cardiovascular disease (CVD) and more severe hepatic fat accumulation [49]. Microsomal TG transfer protein (MTTP) catalyzes the lipidation of ApoB100 and is necessary for the assembly and secretion of VLDL. In MASLD mice, MTTP overexpression effectively reduces triglyceride levels in hepatocytes [50].

## Treatment of MASLD from the perspective of metabolic dysfunction

**Fig. 2:**
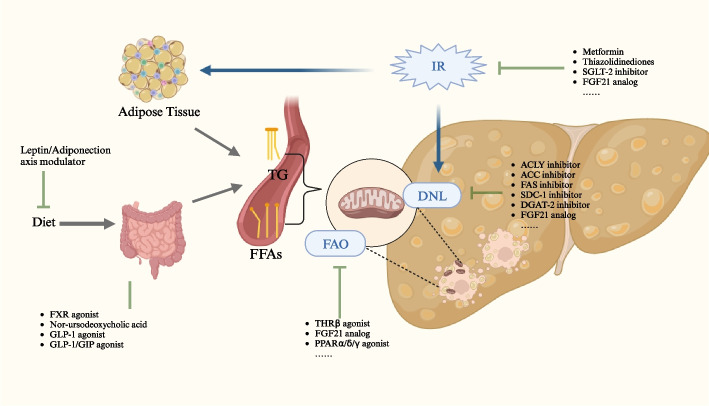
Medications targeting at metabolic dysfunction of MASLD. DNL, de novo lipogenesis; FAO, fatty acid β-oxidation; IR, insulin resistance; GLP, glucagon-like peptide; GIP, glucose-dependent insulinotropic peptide; FXR, farnesoid X receptor; FGF, fibroblast growth factor; TG, triglycerides; FFA, free fatty acid; THR, thyroid hormone receptor; PPAR, proliferator-activated receptor

Given the close causal relationship between metabolic disorders and MASLD, therapies targeting systemic glucose and lipid metabolism have shown promising effects. Lifestyle interventions (calorie restriction and physical exercise) have been proven to be effective in treating MASLD [51], but many patients struggle to adhere to the lifestyle intervention programs due to poor long-term compliance. Bariatric surgery has been proven to be powerful tool for sustainable weight loss and great improvement in liver steatosis in patients with MASLD and morbid obesity [52]. However, the majority of patients with MASLD neither accept invasive surgery nor meet the minimum body mass index (BMI) requirements for bariatric surgery. Therefore, an effective medication for the treatment of MASLD is of great clinical significance. Currently, novel medications targeting metabolic disorders have shown promising results for the treatment of MASLD, as illustrated in Fig. 2.

### Insulin sensitizers

#### Thiazolidinediones (TZD)

Thiazolidinediones is a kind of insulin sensitizers with thiazolidinedione ring, which act as potent activators of the nuclear receptor PPARγ. Thiazolidinediones cause decreased liver lipid accumulation and FFA plasma levels by inducing the release of adipokines, encouraging TG storage in adipose tissue, and strengthening the suppressive effect of insulin on lipolysis [53]. As summarized in Table 1, clinical trials of pioglitazone showed significant improvement in IR, liver steatosis and inflammation compared with placebo [54, 55]. Rosiglitazone showed similar a beneficial effect on liver steatosis, but its adverse effects of detrimental weight gain and edema are severe [56]. The mitochondrial pyruvate carrier (MPC) is another target of thiazolidinediones. MPC is responsible for transporting pyruvate from the cytosol across the inner membrane of mitochondrion [57]. MSDC-0602 K, a PPARγ-sparing thiazolidinedione targeting to MPC, ameliorates hepatic steatosis, circulating liver enzymes and insulin sensitivity in phase IIb trials as well as in mouse models [58]. More importantly, MSDC-0602 K tended to have fewer side effects on bone density and mesenchymal stem cell properties in obese mice compared to pioglitazone [59].

**Table 1:** Major clinical trials assessing thiazolidinediones in patients with MASLD

Drug target	Drug name	Study participants	Main Results	References
PPARγ activators	Pioglitazone	247 adults with NASH but without T2DM	Improve liver histology in 34% participants after 96 weeks. Reduce hepatic steatosis and lobular inflammation but not fibrosis	[54]
		55 patients with NASH comfirmed by liver biopsy and impaired glucose tolerance or T2DM	Decrease aspartate aminotransferase (AST) by 40%, alanine aminotransferase (ALT) by 58%, liver fat content (LFC) by 54%, increase hepatic insulin sensitivity by 48%. Improve histological steatosis, ballooning necrosis, inflammation but not fibrosis after 6 months	[55]
	Rosiglitazone	32 patients with histologically proven NASH	Improve liver steatosis in 47% and serum transaminase levels in 38% of participants after 1 year	[56]
	MSDC-0602 K	392 patients with NASH and fibrosis (F1-F3) confirmed by liver biopsy	Improve the result of liver biopsy in 29.8–39.5%. Significantly reduce liver enzymes and NAS scores after 52 weeks	[58]

#### Metformin

Metformin inhibits hepatic gluconeogenesis and improves IR in patients with type 2 diabetes. Previous studies indicated that metformin effectively improves systemic inflammation and insulin sensitivity, and reduces body weight [60]. However, it also increases hepatocyte DNL that contributes to hepatic TG accumulation [61]. Although it is clear that metformin could not improve liver histological steatosis [62], it’s more often used in combination with other medications at present, such as GLP-1 receptor (GLP-1R) agonists, thiazolidinediones or sodium-dependent glucose transporter 2 (SGLT2) inhibitors [63].

### Lipogenesis inhibitors

#### ACLY inhibitor

When excess citrate is available in cells, ACLY catalyzes the conversion of citrate to acetyl-CoA for lipogenesis. Bempedoic acid (BemA, ETC-1002) is a liver-specific ACLY competitive inhibitor that reduces hepatic steatosis through various pathways [64]. In mouse models that recapitulate different stages of the disease, BemA is proved significant reduction of hepatic TG accumulation, as well as genetically modulation of inflammation and fibrosis [65]. Clinical trials have showed positive outcomes on reduction of low-density lipoprotein (LDL) levels and cardiovascular risk [66, 67], while the efficacy among patients with MASLD remains to be studied.

#### ACC inhibitor

The first and committed step in DNL is catalyzed by ACCs, which convert acetyl-CoA to malonyl-CoA. Additionally, malonyl-CoA is a signaling molecule that inhibits FAO. ACC inhibitors have been proved efficacious to improve liver steatosis in animal models [68]. While in clinical trials, firsocostat (GS-0976) showed benefit in the improvement of liver lipid accumulation, stiffness and serum liver enzymes, but also led to an increase in serum triglycerides [69, 70]. Another three-part randomized phase 1 study showed similar efficacy on PF-05221304 [71]. The safety and tolerance of ACC inhibitors might limit their use in clinical practice and still need to be assessed further.

#### FAS inhibitor

FASs catalyze malonyl-CoA, synthesized by ACC, to saturated long-chain fatty acids. In obese mice, it’s demonstrated that the inhibition of FAS improves hepatic steatosis and IR [72]. Denifanstat (TVB-2640), a FAS targeted inhibitor, remarkably reduces hepatic lipid accumulation and serum alanine transaminase (ALT) without significantly increasing circulating triglycerides [73]. Consistently, a phase 2a trial also found that the FAS inhibitor significantly suppressed the lipid accumulation in the liver assessed by magnetic resonance imaging-proton density fat fraction (MRI-PDFF) and serum biomarkers compared to placebo group [74].

#### SCD1 inhibitor

SCD1 functions to convert saturated fatty acids to monounsaturated fatty acids. The activity of SCD1 is increased in patients with MASLD [75]. In mouse models, aramchol (arachidyl-amido cholanoic acid) prevented steatohepatitis and fibrosis by blocking SCD1 and boosting the flow via the transsulfuration pathway, which kept the cellular redox balance stable [76]. Clinical trials among MASLD patients also indicated that hepatic SCD1 inhibitors dose-dependently improved liver steatosis, steatohepatitis and fibrosis, as measured by MRI-PDFF, serum liver enzymes and liver histology [77]. However, other studies found that 12-week aramchol treatment did not reduce LFC measured by MRI-PDFF or stiffness measured by magnetic resonance elastography (MRE) and vibration-controlled transient elastography (VCTE)[78].

#### DGAT2 inhibitor

The last step in DNL is that diacylglycerol acyltransferase (DGAT) catalyzes fatty acyl-CoA to diacylglycerol. Previous studies showed that lower level of DGAT2 expression leads to reduced steatosis in diabetic mice, but hepatocyte damage is exacerbated by lipotoxicity from FFAs [79]. Phase 1 studies indicated that selective DGAT2 inhibitor (PF-06427878) is well tolerated and significantly improves markers of liver function [80]. IONIS-DGAT2Rx is an antisense oligonucleotide inhibitor of DGAT2 expression which prevents LFC in a phase 2 trial [81]. Another DGAT2 inhibitor named ervogastat (PF-06865571) presented similar efficacy on liver steatosis, without serious gastrointestinal adverse events [82]. It's noting worth that co-administration of ACC inhibitor PF-05221304 and DGAT2 inhibitor PF-06865571 has a stacked efficacy and successfully overcomes the obstacle of ACC [83], although ACC inhibitors alone have obvious adverse effects of elevating serum TG and activating SREBP1c. Apart from a decreased likelihood of dose-dependent elevation in serum lipids, the total incidence of adverse events did not rise as PF-05221304 dose increased [83].

Currently, several novel hepatic lipogenesis inhibitors have shown promising effects in treating MASLD. We summarized the major results of these clinical trials in Table 2.

**Table 2:** Major clinical trials assessing lipogenesis inhibitors in patients with MASLD

Drug target	Drug name	Study participants	Main Results	References
**ACC inhibitor**	GS-0976	10 patients with NASH and 10 healthy volunteers	Reduce hepatic DNL by 22%, MRI-PDFF LFC by 6.6%, MRE liver stiffness by 0.3Kpa, serum ALT by 44U/L after 12 weeks. Decrease MRI-PDFF liver lipid accumulation more than 30% in 70% of participants	[69]
		126 patients with more than 8% of LFC by MRI-PDFF and more than 2.5 kPa of stiffness by MRE or biopsy-proven NASH and F1-F3 fibrosis	Decrease MRI-PDFF LFC by more than 30% in 23–48% participants in a dose-dependent mode after 12 weeks. Increase serum TG in 11–13% of participants	[70]
	PF05221304	305 overweight/obese patients with NAFLD (more than 8% LFC by MRI-PDFF) and with more than 2 of 5 risk factors of NAFLD	Reduce MRI-PDFF LFC by 50–65% with PF-05221304 dose ≥ 10 mg QD after 16 weeks	[83]
**FAS inhibitor**	Denifanstat (TVB-2640)	12 obese men with certain metabolic abnormalities	Reduce fasting DNL by up to 90%, and ALT by 15.8% after 10 days	[73]
		99 patients with more than 8% LFC by MRI-PDFF and liver fibrosis of more than 2.5 kPa by MRE or liver biopsy	Reduce LFC by 9.6% in 25 mg group and 28.1% in 50 mg group after 12 weeks	[74]
**SCD1 inhibitor**	Aramchol	247 patients with NASH proved by biopsy results	Achieve NASH resolution in 16.7% of participants and fibrosis improvement without worsening NASH in 29.5% after 52-week treatment. Reduce ALT by 29.1U/L. Liver fat by MRS was not significantly reduced	[77]
		50 patients with HIV-associated NAFLD (more than 5% LFC by MRI-PDFF)	Do not reduce MRI-PDFF LFC or stiffness by MRE and VCTE after 12 weeks	[78]
**DGAT2 inhibitor**	PF-06427878	24 healthy participants in NCT02855177 and 39 healthy participants in NCT 02391623	Reduce MRI-PDFF LFC and serum ALT, AST, alkaline phosphatase and total bilirubin in healthy adults after 14 days	[80]
	IONIS-DGAT2_Rx_	44 patients with BMI of 27-39 kg/m^2^, HbA1c of 7.3–9.5% and LFC by MRI-PDFF ≥ 10%	Reduce 5.2% LFC by MRI-PDFF after 13-week treatment	[81]
**ACC/DGAT2 inhibitor**	PF-05221304 and PF-06865571	99 overweight patients with metabolic syndrome and/or T2DM and more than 5% LFC by MRI-PDFF	Reduce 44.6% LFC by MRI-PDFF and serum TG after 6 weeks	[83]

### Fatty acid oxidation activators

#### Thyroid hormone receptor (THR) β agonists

Mitochondrial dysfunction is involved in the pathophysiology of MASLD, and in patients with steatohepatitis exhibit decreased activity of respiratory chain complexes and fatty acid oxidation [84]. It is an appealing therapeutic target for MASLD to stimulate mitochondria function according to recent studies [85]. The thyroid hormone receptor consists of 2 isoforms, namely THRα and THRβ. The THR mediates important functions for growth and metabolism at the transcriptional and post-translational levels and via autophagy [86]. THRβ increases hepatic FAO and reduces liver steatosis in rodent models of liver steatosis [87]. Resmetirom (MGL-3196) is a liver-targeted selective THRβ agonist. It resulted in significant reduction in hepatic steatosis and serum lipid metabolic products such as LDL, TG and ApoB and improvement in MRI-PDFF among patients with MASLD, with adverse events of transient mild diarrhoea and nausea [88]. Recently, the published phase 3 trials showed that resmetirom significantly improved liver fibrosis and inflammation, and reduced LFC as well as serum LDL-c, ApoB, and TG concentrations [89, 90].

#### PPAR α/γ/δ agonists

The PPAR transcription factors (PPARα, PPARδ and PPARγ) regulate lipid metabolism through gene transcription. PPARα and PPARδ involve in mitochondrial biogenesis and FAO, as well as fatty acid uptake and TG turnover [91]. PPAR agonists, such as lanifibranor (IVA337), elafibranor (GFT505) and saroglitazar, improved hepatic steatosis, inflammation and fibrosis in MASLD animal models [92]. The pan-PPAR agonist lanifibranor, which acts on three different PPAR isotypes, significantly improves hepatic steatosis, ballooning and inflammation with a relatively low coincidence of adverse events in phase 2b trials [93]. Elafibranor, a co-agonist of PPARα and PPARδ, improved liver enzymes, lipid and glucose metabolism, and systemic inflammation markers in adults and reduced of ALT in children with MASLD, while the efficacy on histological endpoints of liver steatosis remains to be studied [94]. While saroglitazar, another dual PPARα/γ agonist, could also effectively improve LFC assessed by MRI-PDFF and several metabolic parameters [95].

### Incretins and intestinal FXR agonists

#### GLP-1 modulators

GLP-1 is an endogenous intestinal hormone that can lower food intake and peripheral fat mobilization by promoting the synthesis and release of insulin and preventing the secretion of glucagon. It does this by acting through the G protein-coupled GLP-1R. In studies on obese diabetic mice, rats, and rhesus monkeys, GLP-1R agonists enhanced indicators of hepatic steatosis and liver damage [96]. In patients with MASLD, GLP-1R agonists, including dulaglutide [97], exenatide [98], liraglutide [99] or semaglutide [100], have shown beneficial effects on hepatic fat content and liver histological inflammation and fibrosis, as listed in Table 3. Compared with liraglutide and dulaglutide, semaglutide has more pronounced effects on reducing body weight and blood glucose, while dulaglutide has less gastrointestinal symptoms [101, 102]. The benefit of GLP-1R agonists is strongly associated with weight loss. Combination of glucose-dependent insulinotropic peptide (GIP) or glucagon (GCG) with GLP-1R enhances the anti-obesity effect [103, 104]. In high-fat diet-fed mice, GIP increases the activity of feeding centers in hypothalamic, which leads to weight loss and less food intake [105]. Meanwhile, GIP reduces GLP-1R-mediated adverse gastrointestinal events [106]. While GCG increases energy consumption and ameliorates overnutrition and excessive fat accumulation [107]. In patients with obesity and diabetes mellitus type 2 (T2DM), GLP-1/GIP co-agonist tirzepatide showed a significant reduction in body weight, as well as LFC [108] and liver inflammation and fibrosis biomarkers [109]. More recently, a triple agonist retatrutide showed clinically meaningful improvements in patients with obesity or T2DM [110], but its effect on MASLD still requires further investigation.

**Table 3:** Major clinical trials assessing incretins in patients with MASLD

Drug target	Drug name	Study participants	Main Results	References
**GLP-1R agonists**	dulaglutide	64 adults with T2DM and more than 6.0% LFC by MRI-PDFF	Reduce absolute LFC by 3.5% and liver enzyme after 24 weeks	[97]
	Exenatide	76 overweight/obese patients with T2DM and LFC by MRS ≥ 10.0%	Reduce LFC by 17.55U/L and fibrosis 4 score by 0.10 after 24-week treatment	[98]
	Liraglutide	52 overweight patients with NASH proved by biopsy	Achieve resolution in 39% participants and reduce progression of fibrosis (9% vs 36% in the control group) after 48 weeks	[99]
	Semaglutide	320 patients with NASH and liver fibrosis (F1-F3) proved by biopsy	Achieve NASH resolution without worsening fibrosis in 36–59% participants after 72-week intervention. Improve liver fibrosis in 43% participants in the 0.4-mg group	[100]
**GLP-1R/GIP co-agonist**	Tirzepatide	296 overweight/obese participants with T2DM and fatty liver index of at least 60	Reduce absolute MRI-PDFF LFC by 8.09% after 52-week intervention	[108]
		316 patients with type 2 diabetes with or without stable metformin therapy	Reduce ALT, AST, keratin-18 and procollagen III after 26 weeks	[109]

#### FXR agonist

FXR is a bile acid receptor that mediates lipid signaling and reduces blood levels of glucose and lipids in mice [111]. Multi-center clinical trial showed obeticholic acid (OCA), an FXR agonist, can improve the fibrosis and inflammatory activity of liver [112]. However, OCA therapy has a negative impact on serum lipoprotein profile, that increases VLDL and LDL and reduces HDL [113]. Secondary analysis of FLINT trials demonstrated the correlation between 30% relative reduction in MRI-PDFF and histologic improvements such as steatosis and ballooning [114, 115]. Currently, several new-type FXR agonists are also undergoing clinical trials for MASLD. Tropifexor (LJN452) and nonsteroidal cilofexor (GS-9674) led to reduction of liver biochemistry and hepatic steatosis in patients with MASLD compared to placebo [116, 117]. Vonafexor and structurally optimized FXR agonist, MET409 showed similar reduction [118, 119].

### Fibroblast growth factor (FGF) analogues

#### FGF19

At the intersection of the gut, liver, brain, and white adipose tissue, FGF19 is a gastrointestinal hormone that controls the synthesis of bile acid and acts as a transversal metabolic coordinator. Dysregulation of FGF19 may be linked to illnesses that impact lipid metabolism and the gut-liver axis. [120]. FGF19 analogue aldafermin (NGM 282) reduced liver fat and produced a trend toward fibrosis improvement in phase 2 trials with a generally good tolerance [121–123]. Furthermore, elevation of cholesterol associated with aldafermin can be effectively overcome through the co-administration with rosuvastatin, which is considered a reasonable strategy to optimize the cardiovascular risk [124].

#### FGF21

FGF21 acts as a hormone to enhance energy expenditure, diminish the DNL associated enzymes and regulate food preference, though patients with MASLD tend to have elevated FGF21 inversely correlated with IR [125]. High doses of recombinant FGF21 effectively decreased liver lipid content in obese mice and rhesus macaques [126]. A long-acting Fc-FGF21 fusion protein efruxifermin [127, 128] and recombinant FGF21 analog pegozafermin [129, 130] and pegbelfermin (BMS-986036) [131] have shown benefits in reducing hepatic steatosis and inflammation grades in clinical trials, all of which are well tolerated, with acceptable coincidence of diarrhoea and nausea.

### Others targeting at whole-body energy balance

#### Modular of Leptin/Adiponectin axis

Leptin and adiponectin are secreted by white adipose tissue. When overnourished, serum levels of the two adipokine are elevated, which inhibit food intake and accelerate lipid metabolism. Adiponectin combats hepatic steatosis by activating the AMPK pathway to inhibit DNL and activating the PPAR-α pathway to promote FAO, while improves IR in the liver by activating glucose transporter proteins and inhibiting key enzymes of gluconeogenesis [132]. Recombinant leptin, metreleptin showed efficacy among patients with MASLD associated with relative leptin deficiency and partial lipodystrophy, which was considered by stimulating hepatic VLDL-TG secretion through brain-vagus-liver axis according to a recent study [133].

#### SGLT2 inhibitor

SGLT2 is responsible for more than 90% of filtered glucose reabsorption [134]. In a mouse model of T2DM, SGLT2 inhibitor significantly improved liver steatosis and fibrosis [135]. Clinical trials with canagliflozin [136], dapagliflozin [137], and ipragliflozin [138] showed consistent effect in reducing LFC and liver histological steatosis. Because SLGT2 is not expressed in the liver, weight loss brought on by treatment and improvements in IR may cause a decrease in liver steatosis [134].

## Conclusion

MASLD is a global health problem with no medications licensed for its treatment currently. Due to the close association between metabolic dysfunction and MASLD, many medications targeting at hepatic lipid and glucose metabolism have shown promising results in patients with liver steatosis and metabolic disorders. The summary of the pathogenesis and latest medications of MASLD in this review will help physicians and researchers update the latest achievements in the field. The new nomenclature of MASLD strictly divides the patients with liver steatosis into groups according to the presence of metabolic dysfunction, and can remarkably reduce the heterogeneity of NAFLD. Further well-designed clinical trials are still required to evaluate the possibility and efficacy to treat patients with MASLD by targeting their common metabolic dysfunction. Additionally, MASLD is still a heterogeneous disease with complex and multiple causes [1]. Therefore, with understanding of the heterogeneity of MASLD, a proper clinical classification of MASLD may facilitate the choice of medications for every patient with MASLD. More importantly, since MASLD is a complex phenotype shaped by the dynamic interaction of multiple risk factors, including genetic predisposition, environmental factors and metabolic disorders, a combination of medications targeting at different steps of the pathogenesis of MASLD may achieve optimal therapeutic effect in the future.

## Data Availability

No datasets were generated or analysed during the current study.
